# Prevalence of anti-lymphocyte IgM autoantibodies driving complement activation in COVID-19 patients

**DOI:** 10.3389/fimmu.2024.1352330

**Published:** 2024-04-17

**Authors:** Ainhoa Pérez-Díez, Xiangdong Liu, Stephanie Calderon, Ashlynn Bennett, Andrea Lisco, Anela Kellog, Frances Galindo, Matthew J. Memoli, Joseph M. Rocco, Brian P. Epling, Elizabeth Laidlaw, Mike C. Sneller, Maura Manion, Glenn W. Wortmann, Rita Poon, Princy Kumar, Irini Sereti

**Affiliations:** ^1^ Laboratory of Immunoregulation, National Institute of Allergy and Infectious Diseases (NIAID), NIH, Bethesda, MD, United States; ^2^ Division of Clinical Research, NIAID, NIH, Bethesda, MD, United States; ^3^ Laboratory of Infectious Diseases, National Institute of Allergy and Infectious Diseases (NIAID), NIH, Bethesda, MD, United States; ^4^ Section of Infectious Diseases, MedStar Washington Hospital Center, Washington, DC, United States; ^5^ Division of Hospital Medicine, Georgetown University Medical Center, Washington, DC, United States; ^6^ Division of Infectious Diseases and Tropical Medicine, Georgetown University Medical Center, Washington, DC, United States

**Keywords:** COVID-19, autoantibodies, complement deposition, anti-lymphocyte Ab, lymphopenia, convalescent COVID-19, complement activation, complement-dependent cytotoxicity

## Abstract

**Introduction:**

COVID-19 patients can develop autoantibodies against a variety of secreted and membrane proteins, including some expressed on lymphocytes. However, it is unclear what proportion of patients might develop anti-lymphocyte antibodies (ALAb) and what functional relevance they might have.

**Methods:**

We evaluated the presence and lytic function of ALAb in the sera of a cohort of 85 COVID-19 patients (68 unvaccinated and 17 vaccinated) assigned to mild (N=63), or moderate/severe disease (N=22) groups. Thirty-seven patients were followed-up after recovery. We also analyzed in vivo complement deposition on COVID-19 patients’ lymphocytes and examined its correlation with lymphocyte numbers during acute disease.

**Results:**

Compared with healthy donors (HD), patients had an increased prevalence of IgM ALAb, which was significantly higher in moderate/severe disease patients and persisted after recovery. Sera from IgM ALAb+ patients exhibited complement-dependent cytotoxicity (CDC) against HD lymphocytes. Complement protein C3b deposition on patients’ CD4 T cells was inversely correlated with CD4 T cell numbers. This correlation was stronger in moderate/severe disease patients.

**Discussion:**

IgM ALAb and complement activation against lymphocytes may contribute to the acute lymphopenia observed in COVID-19 patients.

## Introduction

SARS-CoV-2 infects human cells through the angiotensin-converting enzyme 2 (ACE-2) receptor, which is restricted to type 2 lung epithelial cells and, at lower levels, endothelial and epithelial cells from several tissues (1), causing COVID-19 (2, 3). Hospitalized COVID-19 patients with severe disease present a broad pathology, characterized by general inflammation (4) and tissue damage in several organs, such as the lung, brain, kidney, heart, and liver. These varied symptoms are, to some extent, caused by coagulopathy (5), and endothelitis (6) and seem to be immune-related (7), with complement (8, 9) and inflammasome activation playing important roles (10, 11). The beneficial effects of corticosteroid treatment (12) further support the potential autoimmune role of this severe and variable pathology. Indeed, a few groups have identified autoantibodies (AAb) with broad specificity in the sera or plasma of hospitalized COVID patients (13–19). The AAb reactivity spectrum is comparable, if not higher, to that observed in well-characterized autoimmune diseases such as systemic lupus erythematosus (SLE), and includes membrane proteins expressed on lymphocytes (13, 20). The presence of anti-lymphocyte antibodies (ALAb) might play an important role in disease outcome, and more specifically in the lymphopenia observed in most COVID-19 patients, characterized by low numbers of CD4, CD8, and, to a lesser extent, B cells (21, 22). However, the frequency, characteristics, and potential cytotoxic role of ALAb in COVID-19 patients have not been systematically addressed.

We previously found that patients with idiopathic CD4 lymphopenia (ICL) harbor AAb of broad specificities, some of which target membrane proteins on T lymphocytes (23). ALAb-induced lytic activity against T cells by complement and/or NK cell-mediated lysis, and the presence of antibodies with lytic activity in plasma from ICL patients was correlated with more severe lymphopenia (23). Since, similar to ICL, COVID-19 patients generate ALAb and develop (if transient) lymphopenia systematically characterized the frequency, isotype, and potential lytic activity of ALAb in COVID-19 patients. We analyzed the plasma from 85 acutely infected COVID-19 patients with mild or moderate/severe infection, 17 of which were mild breakthrough (after vaccination) cases, for the presence of ALAb, their potential lytic function, and *in vivo* complement activation, with longitudinal patient testing after recovery when available. We found that COVID-19 patients, especially moderate/severe patients, had IgM ALAb at the time of diagnosis and remained detectable during convalescence. IgM ALAb was also found in mild cases, independent of vaccination status and could induce complement-dependent cytotoxicity (CDC). In addition, complement deposition was observed in patients’ lymphocytes, especially in unvaccinated patients, where it was inversely correlated with the patients’ CD4 T cell numbers. Together, our data suggest that IgM ALAb and complement activation might significantly contribute to the lymphopenia observed during COVID-19 acute infection.

## Materials and methods

### COVID-19, influenza patients, and healthy donor (HD) study participants

COVID-19 patients provided PBMC and plasma through enrollment in the clinical protocol “COVID-19-associated Lymphopenia Pathogenesis Study in Blood (CALYPSO)” (NCT04401436). PBMC and sera from influenza were provided from donors enrolled in NCT01646138 “Influenza A 2009 H1N1 Challenge Study in Healthy Adults” (24). The HD provided PBMC, sera, and plasma through the National Institutes of Health (NIH) blood bank. Eligible COVID-19 participants were adults with confirmed COVID-19 by PCR. Patients were assigned to either the mild group, with no or mild symptoms requiring oxygen<4 L through a nasal cannula (NC), or the moderate/severe group, with oxygen requirements >4 L through NC or non-invasive or mechanical ventilation. Patients that had recently (1–3 months) recovered per Centers for Disease Control (CDC) guidelines were assigned to the convalescent group. For long-term convalescent analysis, we utilized plasma from patients enrolled in the clinical protocol “A Longitudinal Study of COVID-19 Sequelae and Immunity” (NCT04411147) (25).

### Flow cytometry

Cells were blocked with anti-human CD16/32 antibody (BD Biosciences) for 10 minutes (min) before adding fluorochrome-conjugated antibodies (**Supplementary Table S1**). For *ex vivo* complement deposition staining, PBMC were incubated at room temperature for 45 min in Gelatin-Veronal buffer (GVB) (CompTech Complement Technology Inc.) before continuing with regular staining. We used LIVE/DEAD (L/D) fixable Dead Cell Stain kits for blue, aqua, or near-IR (Invitrogen) to exclude dead cells. Cells were then incubated for 30 min at room temperature and washed once with 1% BSA-PBS before acquisition using Fortessa (BD Biosciences). The analysis was performed using FlowJo (Tree Star) software (version 10.7 and above).

### Detection of anti-lymphocyte antibodies by flow cytometry

After blocking with anti-human CD16/32 antibody (BD Bioscience), two million HD PBMC were incubated for 30 min at room temperature (RT) in 50 µL of HD, COVID-19, or influenza patient plasma or serum. Plasma or serum pooled from at least 10 HD (HDp) was run on every assay, and its MFI was used to normalize the MFI of individual samples. Cells were washed three times with 1% BSA-PBS and stained with fluorescently labeled anti-human IgG and IgM antibodies (**Supplementary Table S1**) to detect the presence of ALAb attached to CD4+, CD8+, or CD19+ lymphocytes. For each Ig channel and cell population we calculated the normalized MFI (nMFI) by dividing the MFI of each sample by the HDp MFI. Positive detection of ALAb was determined when the nMFI of the sample was ≥3 × SD + mean nMFI of the 20 HD.

### Complement dependent cytotoxicity

HD PBMCs were divided into two halves: the first half was labeled with 50 nM CFSE for 10 min at RT followed by incubation for 30 min at RT with one of the following conditions: CM, HDp, plasma, or serum from individual patients, anti-human CD4 Ab (clone M-T477, BD Biosciences), or anti-human CD20 Ab (rituximab). The second half was incubated with human AB serum (HAB) and used as a CFSE-negative internal control. After washing with CM, equal numbers of CFSE-negative and CFSE-positive PBMCs were mixed under each test condition. The sample originating from mixing CFSE-negative and CM-incubated CFSE-positive cells was placed at 4°C until it was ready to be stained for flow cytometry and was the “ratio tube” to determine the initial CFSEneg/CFSEpos ratio. After washing once with CM, the remaining mixed tubes were resuspended in a 1:4 dilution of freshly reconstituted rabbit complement (Cedarlane) and incubated at 37°C for 1 h. After washing with 1% BSA-PBS, all samples, including the ratio tube, were stained, fixed in 1% PFA, and analyzed by flow cytometry. The percentage of CDC for each lymphocyte population was calculated using the following formula:


[1−(# cells CFSEneg/# cells CFSEpos) ratio tube/(# cells CFSEneg/# cells CFSEpos) test] × 100

### Immunoglobulin depletion from plasma

To deplete total immunoglobulins from patient plasma, a Pierce Thiophilic Adsorption Kit (Thermo Fisher Scientific) was used following the manufacturer’s instructions. Briefly, 200 μL of patient plasma was diluted in 3 mL of binding buffer and loaded into the thiophilic columns after equilibration four times with 3 mL binding buffer. The samples were allowed to completely enter the resin bed before the columns were washed three times with 3 mL binding buffer. A total of 12 mL of flow-through was collected as the Ig-depleted plasma. Three kDa MWCO Amicon Ultra-15 centrifugal filter units (Millipore Sigma) were used for buffer exchange the Ig-depleted fraction by spinning at 3,000 rpm for 60 min for eight exchanges. After the last wash, the Ig-depleted plasma was resuspended in 200 μL of PBS containing 10% of HCpool plasma.

### NK-mediated antibody dependent cellular cytotoxicity

Using the EasySep™ Human NK Cell Isolation Kit, NK cells were negatively selected from HD PBMC and incubated in CM with 1,000 IU/mL of rhIL-2 (Peprotech) at 37°C overnight. Autologous PBMCs were incubated in CM at 37°C overnight and used the following day as target cells after counting and splitting them into two halves. One half was labeled with 5 nM CFSE and incubated with 50 µL of HDp or COVID-19 patient plasma for 30 min at RT. The other half was labeled with 50 nM of CFSE and incubated with CM for 30 min at RT. After washing once with PBS, the same numbers of high- and low-CFSE-labeled target cells were combined and washed again. These target cells were plated in duplicate at 10,000 cells/well in a U bottom 96 well plate. Autologous NK cells were added at the described E:T ratios, except for two control wells for each plasma donor, where no NK cells were added. These control wells were used to calculate the expected ratio of CFSE-low to CFSE-high targets when there was no killing. After 4 h of incubation at 37°C, cells were harvested and stained for flow cytometry. The percentage of killing was calculated based on the number of cells in the CFSE low versus the CFSE high gates in each well and the population of interest using the following formula: 100 − [(# cells CFSEhigh/# cells CFSElow) average control wells/(# cells CFSEhigh/# cells CFSElow) average at a specific ratio] × 100. Anti-CD20 antibody (rituximab) was used as a positive control, and only experiments in which rituximab-induced ADCC on B cells were included.

### Plasma C5a concentration

We measured the C5a concentration in the plasma of patients using ELISA from Meso Scale Discovery Platforms, according to the manufacturer’s instructions.

### Statistics

Two-tailed non-parametric tests were used to compare the differences between groups and correlations. GraphPad Prism (version 9 and above) was used for the statistical tests. A two-tailed *P*-value less than 0.05 was considered significant and adjustment for multiple comparisons was performed as reported. Experiment-specific statistical methods are described in the figure legends.

### Ethics statement

This study was approved by the Institutional Review Board of the National Institutes of Health (NIH). Written informed consent was obtained from all participants prior to any study procedure, in accordance with the Declaration of Helsinki.

## Results

### Characteristics of COVID-19 patients

Patient characteristics are described in **Table 1**. Their median age was 55 years and both sexes were equally distributed. Among the 85 acutely infected and mostly hospitalized patients, 46 mild cases and 22 moderate/severe cases were unvaccinated individuals enrolled from May 2020 to April 2021, whereas 17 mild cases were breakthrough infections enrolled between April 2021 and June 2022. We also obtained convalescent samples from 37 patients at a median of 58 days (range, 20–259 days) after their acute sampling. As controls, we analyzed 20 HD and seven influenza-infected individuals. The median age was similar between HD and COVID-19 patients, and the influenza-infected volunteers were younger. The latter group had samples collected at a median of three days after symptom onset (DSO), while we collected COVID-19 samples at a median of nine DSO.

**Table 1:** Characteristics of COVID-19 patients.

Disease Status	Total (N)	Breakthrough (N)	Age^a^ (years)	Sex (% males)	CD3^a^ (cells/μL)	B cell^a^ (cells/μL)	DSO^a,b^
**Mild**	63	17	52 (38–62)	54	941 (654–1,149)	130 (88–216)	9 (6–13)
**Mod + Sev**	22	0	56 (48–67)	50	803 (478–1,424)	190 (87–333)	10 (8–15)
**All acute**	85	17	55 (42–63)	52	920 (639–1,155)	145 (87–234)	9 (6–13)
**Convalescent**	37	17	51 (35–56)	41	1,209 (1,030–1,678)	188 (149–246)	66 (49–82)
**Flu**	7	0	32 (26–33)	14	Not available	Not available	3 (3–4)
**HD**	20	Not apply	53 (35–58)	80	Not available	Not available	Not apply
p-value^c^			0.0013^d^	0.4180	0.0006^e^	0.0537	0.0006^f^

Median days between acute and convalescent samples is 58 (IQR range 39-70).

a Medians (IQR).

b Days post symptom onset.

c Groups were compared using Kruskal–Wallis test followed by Dunn’s multiple comparisons test, except when looking at sex differences, where we used Fisher’s exact test. The group “all acute” was not included in the comparisons because its patients were included in mild or Mod + Sev groups.

d Flu group was significantly different from the Mild and Mod + Sev groups.

e Convalescent group was significantly different from the Mild and Mod + Sev groups.

f Convalescent group was not included in the comparisons. Flu group was significantly different from the Mild and Mod + Sev groups.

### Prevalence of IgM anti-lymphocyte antibodies in people with COVID-19

**Figure 1:**
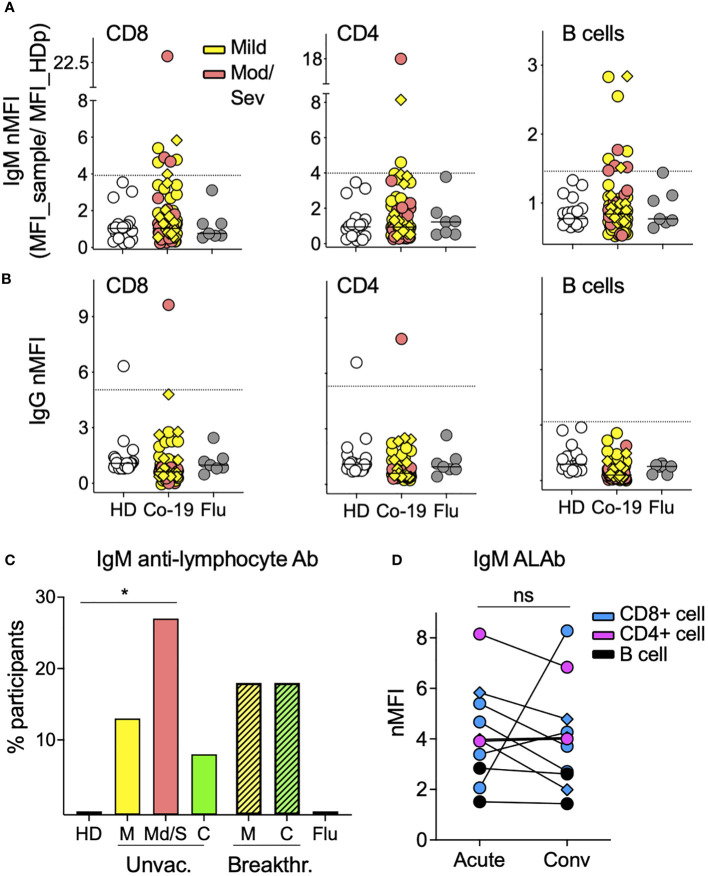
The prevalence of IgM antilymphocyte antibodies in COVID-19 patients. **(A)** IgM and **(B)** IgG anti-lymphocyte antibodies (ALAbs), that bind CD8 (left), CD4 (middle), or B cells (right) were measured in plasma or sera, as described in *Materials and methods* (see also **Supplementary Figure S1**), from 85 COVID-19 patients (63 mild and 22 moderate/severe, in yellow and orange symbols, respectively), 20 HD in open symbols and seven volunteers infected with flu four days earlier (gray circles). Each symbol represents the normalized Mean Fluorescent Intensity (nMFI) of individual samples, calculated by dividing each sample’s MFI by the MFI of the same HD pool (HDp) run in each experiment. Samples from breakthrough COVID-19 patients are represented by diamonds. HD and Flu nMFIs are the means obtained from two experiments that tested two different HD PBMC as targets. For each lymphocyte population, the discontinuous line represents the HD mean nMFI plus three times their SD, and nMFI equal to or above this threshold was considered positive. **(C)** Percentage of participants with IgM Ab against at least one of the three lymphocyte populations shown in A in 20 HD, seven Flu patients, 68 unvaccinated COVID-19 patients [46 mild (M), 22 moderate/severe (Mod/Sev) and 20 convalescent **(C)**], and 17 breakthrough COVID-19 patients (all mild and convalescent pairs). *p = 0.0216 by two-sided Fisher’s exact test. **(D)** IgM nMFI in plasma or sera from COVID-19 acute and convalescent longitudinal samples. Only patients with both time points available and with IgM ALAb against either CD8 T cells (blue), CD4 T cells (pink), or B cells (black) in at least one of the two time points are displayed. Ns, no significant difference by two-tailed Wilcoxon test.

Using a previously described flow-based assay (23), we screened the plasma from 85 acutely infected COVID-19 patients (68 unvaccinated and 17 breakthrough cases) for the presence of ALAb. Plasma samples with IgM or IgG normalized mean fluorescence intensity (nMFI) signals ≥3 times the SD above the mean nMFI of the 20 HD were identified as positive (example shown in **Supplementary Figure S1**). We found that 21% (15 of 85) of COVID-19 patients had IgM ALAb at the time of diagnosis, while none were found in either HD- or influenza (flu)-challenged and infected individuals (24) (**Figures 1A, C**). We found no increase in IgG ALAb levels, with only one patient showing strong anti-T cell IgG reactivity (**Figure 1B**). The proportion of patients with IgM ALAb was significantly higher in moderate/severe (Mod/Sev) patients than in HD patients (p = 0.0216), and approximately double that in unvaccinated mild patients (27% and 14%, respectively) (**Figures 1A, C**). Vaccination status did not significantly alter IgM autoreactivity in mild cases, with 18% of breakthrough patients showing IgM ALAb (**Figure 1C**). Since the acute samples were taken at a median of nine days after symptom onset (**Table 1**), we next asked if the prevalence and isotype of ALAb changed during convalescence. For this, we retested 37 patients during their early convalescence period, which averaged 2 months after their positive test (**Table 1**) and found a similar prevalence of IgM ALAb to their acute samples in both unvaccinated and breakthrough cases (**Figure 1C**). Furthermore, the IgM ALAb levels found in individual acute patients remained mostly unchanged at their convalescence time points (**Figure 1D**). Except for a patient who was categorized as mild at the time of acute sample collection but later developed severe symptoms. Blood B-type antigens have been described to be present on lymphocyte membranes and targeted by IgG antibodies in 7% of mismatched kidney transplant recipients (26). To rule out the possibility that the IgM ALAb detected in COVID-19 patients were directed against ABO antigens, we re-tested, this time against type O PBMC, 10 HD plasma samples and 20 COVID-19 samples previously found to be IgM ALAb positive (n=16) or negative (n=10) for IgM ALAb, against type 0 PBMC. Using type O PBMC, we obtained almost identical results as previously observed when using untyped PBMC (**Supplementary Figure S2**). We conclude that a significant proportion of COVID-19 patients, independently of vaccination status, harbor IgM ALAb that is maintained during early convalescence.

Because the convalescent time point we analyzed was relatively early after acute infection, we next screened for ALAb in a second cohort (N = 15) of mild non-hospitalized COVID-19 patients (25) at six to 12 months after their positive PCR test. When compared to their respective HD control groups, we found no IgM or IgG ALAb (**Supplementary Figure S3**), suggesting that IgM ALAb may not prevail for more than 6 months in mild cases of non-hospitalized COVID-19, although our sample size may have been too small to detect residual autoantibodies, especially in these mild non-hospitalized cases.

### Anti-lymphocyte antibodies in people with COVID-19 can exert cytotoxicity

**Figure 2:**
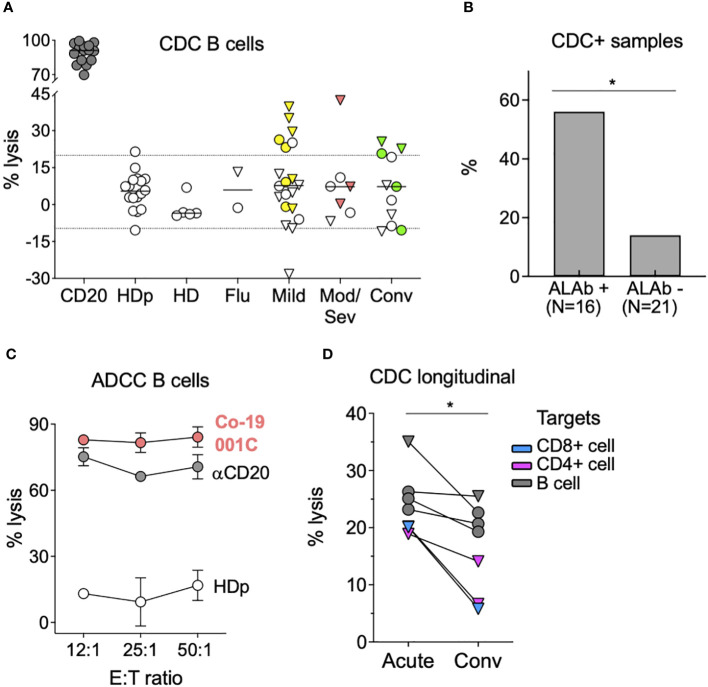
Anti-lymphocyte antibodies in patients with COVID-19 exert cytotoxicity. **(A)** Complement-dependent cytotoxicity (CDC) tested in plasma or sera samples included in **Figure 1**, comprising 38 COVID-19 participants (20 mild, seven moderate/severe, and 11 convalescents), five HD, and two flu patients. CDC was tested, as described in the *Materials and methods*, with B cells as targets and anti-CD20 Ab as a positive control. A total of 17 independent experiments were performed, each testing the same HDp sample. Values from individual experiments are represented by circles, and triangles represent the median values from two to four replicate experiments. Yellow, salmon, and green symbols represent mild, mod/sev, and convalescent IgM ALAb positive samples, respectively. Discontinuous lines represent the mean ± 2SD of the HDp samples. See also **Supplementary Figure S4A**. **(B)** Plasma samples from COVID-19 participants tested for CDC in A were divided based on the presence or absence of ALAb, with 16 and 21 samples respectively, and the percentage of samples with CDC activity against either lymphocyte (CD4, CD8, or B cells) in each group was calculated. *p = 0.0124 by two-sided Fisher’s exact test. **(C)** Antibody-dependent cellular cytotoxicity (ADCC) against B cells triggered by plasma from moderate/severe patient (Co-19 001C, in salmon) with high levels of IgG ALAb, as shown in **Figure 1B**. White and gray circles represent HDp and anti-CD20 antibodies diluted in HDp, respectively. Three different NK effectors-to-target (E:T) ratios were tested. Circles represent the mean, and bars represent the SD of duplicates. A representative experiment out of five is shown. See also **Supplementary Figure S4C**. **(D)** The percentage of CDC lysis triggered by plasma from COVID-19 acute and convalescent longitudinal samples. Only patients with both time points available and with CDC activity against either CD8 T cells (blue), CD4 T cells (pink), or B cells (black) at least in one of the two time points are shown. Values from individual experiments are represented by circles, and triangles represent the median values from two to four replicate experiments. *p<0.05, two-tailed Wilcoxon test.

We next investigated whether the ALAb observed in COVID-19 plasma could exert lytic activity against HD lymphocytes. For this, we tested plasma from 37 COVID-19 samples with (n = 16) or without (n = 21) IgM ALAb, five HD, and two flu-infected individuals for their ability to induce CDC of HD CD4+, CD8+, or B lymphocytes. We found CDC activity, mostly against B cells, in nine and three COVID-19 samples with and without IgM ALAb, respectively (**Figure 2A**; **Supplementary Figure S4A**). CDC activity was detected in plasma from mild, moderate/severe, and breakthrough patients, while none was detected in HD and flu samples. The incidence of CDC was statistically higher in COVID-19 samples with IgM ALAb than in samples without IgM ALAb (56% and 14%, respectively) (**Figure 2B**). CDC was dependent on immunoglobulins, presumably IgM, since the CDC activity of IgM ALAb+ samples was lost after Ig depletion (**Supplementary Figure S4B**). Notably, plasma from the only patient with IgG ALAb, and who also had IgM ALAb, displayed both CDC and very strong ADCC against B, CD4+ and CD8+ lymphocytes (**Figure 2C**; **Supplementary Figure S4C**). Next, we asked whether the CDC found in the patients’ plasma at the acute time point was maintained during their convalescent period in those with sample availability. Although most patients maintained a positive CDC at the convalescent time point, the level was significantly lower (p = 0.0156) (**Figure 2D**). Thus, we found that plasma from around half of the COVID-19 patients with ALAb possesses CDC against mostly B cells, independent of disease severity or vaccination status, and that this CDC, although still detectable, diminishes during convalescence.

### Lymphocytes from COVID-19 patients display *in vivo* complement deposition

**Figure 3:**
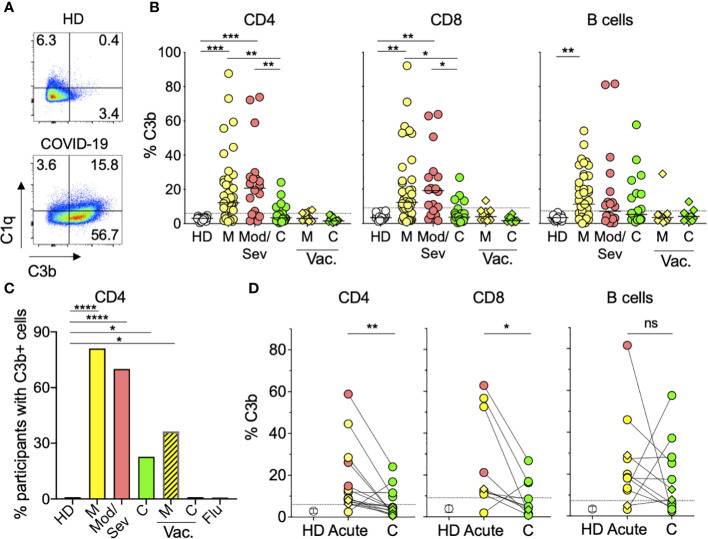
Lymphocytes from COVID-19 patients display *in vivo* complement deposition. **(A)** Representative flow cytometry graphs showing deposition of C1q and C3b classical complement components on the surface of CD4 T cells from an HD patient (top) and an acute mild unvaccinated COVID-19 patient (bottom). PBMC from donors were stained as described in the *Materials and methods*. The numbers represent the percentages of CD4 T cells in each quadrant**. (B)** Percentages of CD4 (left panel), CD8 (middle panel), or B (right panel) cells with C3b deposition. Symbols represent individual PBMC donors from 19 HD (open circles) and 63 unvaccinated COVID-19 patients, of which 43 were mild (M) (yellow circles), 20 were moderate/severe (Mod/Sev) (salmon circles), and 22 were convalescent (C) (green circles). PBMC from 11 breakthrough vaccinated (vac.) cases are shown in diamonds representing mild (M) or convalescent (C) status in yellow and green, respectively. The dotted line represents 3 × SD over the mean of the HD signal, and participants with percentages above this threshold were considered positive for complement deposition and are shown in **(C)** for CD4 T cells. Kruskal–Wallis test followed by Dunn’s test adjusting for multiple (11) comparisons, was performed. *p<0.05; **p<0.01; ***p<0.001. **(C)** Percentage of participants with C3b deposition on their CD4 T cells calculated from the same donors and color coding shown in panel B plus the seven Flu patients and the same time point shown in **Figure 1**. Vaccinated COVID-19 patients (M, C) are represented by the striped bars. *p<0.05; ****p<0.0001 using two-sided Fisher’s exact test. **(D)** Percentage of CD4 (left panel), CD8 (middle panel), or B (right panel) cells with complement deposition from acute and convalescent (C) time points from the same patient when available. Only patients with at least one positive time point are displayed. Open circles represent the mean percentage of C3b in HD cells, and vertical bars represent the standard deviation (SD). Closed circles and diamonds represent cells from the unvaccinated and breakthrough patients, respectively. Ns, not significant; *p<0.05; **p<0.01 using two-tailed Wilcoxon test.

Since complement activation is the main functional feature of IgM Ab, we next asked whether we could detect *in vivo* complement activity in addition to the observed CDC. For this, we directly stained PBMC from 63 COVID-19 patients for the complement subunits C3b and C1q and quantified the percentage of lymphocytes (CD4+, CD8+, or B cells) with complement deposition on their membranes (**Figure 3A**; **Supplementary Figure S5A**). We found both C3b and C1q on the lymphocytes membrane. C3b displayed a larger quantitative range and was significantly increased on CD4 and CD8 T cells from mild and moderate/severe unvaccinated patients compared to HD (**Figures 3A, B**). Overall, approximately 70% of acutely infected unvaccinated patients displayed complement deposition on their CD4 T lymphocytes versus none of the HD or seven flu-infected individuals (**Figure 3C**). Similar levels of C3b were found in naïve and activated T cells (**Supplementary Figure S5B**), suggesting that C3b deposition was not mediated by C3b secretion or binding to its receptor in an autocrine fashion, as previously described for TCR-stimulated CD4 T cells (27). Furthermore, the abovementioned increased levels of C1q deposition on T cells, which is characteristic of the classical complement activation pathway, in contrast to C3b, is not secreted by activated T cells (**Figure 3A**). Although still present, complement deposition on lymphocytes was observed in a lower proportion (23%) of convalescent unvaccinated patients (**Figures 3B, C**) and at the individual sample level, the percentage of T lymphocytes with complement deposition significantly decreased during convalescence (**Figure 3D**). This decrease was not observed in B lymphocytes, in which complement deposition levels were maintained during convalescence (**Figure 3D**). Notably, we observed much lower levels of complement deposition on lymphocytes in breakthrough patients (36%) (**Figure 3C**), which was not significantly different from HD (**Figure 3B**). Our data show that most unvaccinated COVID-19 patients display *in vivo* complement deposition on their lymphocytes during acute infection, recovering to normal levels during convalescence.

### Complement deposition on CD4 T cells inversely correlates with CD4 T cell numbers in COVID-19 patients

**Figure 4:**
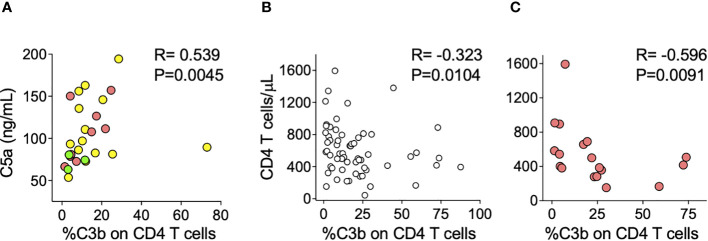
Complement deposition on CD4 T cells correlates with C5a concentration in plasma and CD4 T cell numbers in COVID-19 patients. **(A)** Percentage of CD4 T cells with *in vivo* C3b deposition in 27 unvaccinated COVID-19 patients (14 mild in yellow, 10 Moderate/Severe in salmon, and three convalescents in green) correlates with the concentration of C5a in plasma from the same patients. Longitudinal samples from the same person were excluded. **(B, C)** The percentage of CD4 T cells with *in vivo* C3b deposition inversely correlates with CD4 T cell numbers from either all **(B)** or only moderate/severe **(C)** acute COVID-19 patients. **(A–C)** Each circle corresponds to an individual patient and a two-tailed Spearman correlation was used.

Other groups have described complement activation in COVID-19 patients; specifically, plasma C5a levels have been associated with increased COVID-19 severity and inflammation (8, 28). We wanted to test whether the level of complement deposition detected on lymphocytes of COVID-19 patients might correlate with the C5a concentration in plasma. We measured C5a concentrations in plasma from 19 unvaccinated COVID-19 patients and found a positive correlation with lymphocyte C3b deposition (R = 0.539, P = 0.0045) (**Figure 4A**), further supporting the *in vivo* systemic activation of complement. Notably, C5a plasma levels seemed to correlate better with C3b deposition on CD4 T cells than with disease severity. Since lymphopenia is a known characteristic of COVID-19 acute disease that, similar to C5a, is associated with disease severity (21, 22), we evaluated whether *in vivo* complement deposition on T lymphocytes might have implications on blood T cell numbers. When analyzing all acute COVID-19 patients from whom we had both measurements (N = 62), we found an inverse correlation between the level of complement deposition on CD4 T cells and CD4 T cell numbers (R = −0.323, P = 0.0104) (**Figure 4B**). This correlation was stronger when analyzing the subgroup of moderate/severe patients (R = −0.596, P = 0.0091) (**Figure 4C**), suggesting that *in vivo* complement activation might be implicated in CD4 lymphopenia observed during acute COVID-19.

## Discussion

We systematically analyzed the prevalence and function of ALAb in both hospitalized-unvaccinated and non-hospitalized breakthrough COVID-19 patients and found IgM ALAb in both groups, especially in moderate/severe patients, which was still detectable during convalescence. IgM ALAb displayed lytic activity against HD lymphocytes, mostly against B cells. We also found high levels of *in vivo* complement deposition on lymphocytes from unvaccinated COVID-19 patients, which reverted during convalescence. The level of complement deposition on lymphocytes was significantly correlated with markers of disease severity, such as C5a and lymphopenia. Thus, these data provide evidence of relaxed peripheral B-cell tolerance through IgM autoantibody production and complement activation directed to lymphocytes, with clinical consequences in COVID-19 patients.

Previous studies have described a decline in peripheral B cell tolerance in COVID-19 patients (29, 30) with the presence of IgG autoantibodies directed against a variety of targets expressed in multiple tissues (16, 18, 31, 32), including immune cells and, specifically B cells (13, 20). In some cases, these class-switched autoantibodies were found at the time of diagnosis, suggesting they might have been present before COVID-19 infection. IgM-secreting cells, although not extensively studied, have been shown to be concomitantly expanded with autoimmune-rich IgG1 antigen-secreting cells (ASC) during acute SARS-CoV-2 infection, with both isotypes following similar declining dynamics during convalescence (20). In addition, unswitched IgM autoantibodies of broad specificities were previously detected in more than 90% of critically ill COVID-19 patients (33), suggesting the *de novo* generation of autoantibodies soon after infection. This study did not follow the patients longitudinally to determine whether the autoantibodies remained during convalescence and/or followed class-switch recombination. Our present work shows that IgM driven autoimmunity can activate complements against lymphocytes *in vitro*, suggesting that it might play a role in inducing *in vivo* complement fixation, which correlates with higher lymphopenia during acute disease. The IgM ALAb did not class switch, and their levels were maintained during an early convalescence period of one to three months, which is in agreement with previous studies describing IgG autoreactivity in COVID-19 patients (15, 16, 20, 34). We cannot rule out that these IgM ALAb preexisted in COVID-19 disease patients since we did not have access to pre-infection plasma. Interestingly, when analyzing a different cohort of mostly non-hospitalized COVID-19 patients with a longer convalescent time frame, ranging between 6 and 12 months, ALAb was not detected. Although this latter group was not exactly comparable to our initial cohort, where most of the unvaccinated patients were hospitalized during acute disease, our findings agree with Woodruff and colleagues’ work, where most of the IgG autoantibodies decreased or disappeared 6-12 months after acute infection (20). IgM ALAb has been previously described as part of the polyreactive natural IgM autoantibodies produced by B1 lymphocytes that are found at low levels in HD and increase during infections (35). These natural IgM autoantibodies are not able to activate complement at 37°C and are thought to play a protective role against inflammation. Although a large proportion of the IgM ALAb described here were able to induce *in vitro* lymphocyte lysis by CDC (which is performed at 37°C), we cannot rule out the possibility that some of them could be related to the previously described natural IgM autoantibodies.

Since the main function of the IgM isotype is to activate the classical complement cascade, it is perhaps not surprising that we found both CDC activity directed to lymphocytes in patients’ plasma, as well as complement deposition on COVID-19 patients’ T cells directly *ex vivo*. Our group (36) and others (9) have previously shown complement activation in COVID-19 patients, and C5a concentration in plasma has been associated with disease severity (28). However, the potential link between autoantibodies and complement activation in COVID-19 patients has not yet been examined. Although pathways other than IgM ALAb might induce complement deposition on T cells (37), we show here that CDC activity found in COVID-19 plasma samples is immunoglobulin dependent and that patients with IgM ALAb have significantly increased CDC against lymphocytes, suggesting a potential *in vivo* link between IgM ALAb and complement activity in patients’ lymphocytes. The presence of C3b on *ex vivo* lymphocytes might also be a consequence of intracellular C3 cleavage in antigen-stimulated T cells, followed by C3b secretion and binding to its receptor, CD46, in an autocrine manner (27, 38). We ruled out this possibility after finding similar levels of C3b deposition on T cells independent of their activation status, although the amplitude of the *ex vivo* C3b deposition we found on COVID-19 lymphocytes might be partially explained by CD46 binding to CD3b secreted by other cells, such as tissue activated T cells and/or other hematopoietic and non-hematopoietic cells (27), specifically during COVID-19 infection (39). We also found C1q deposition in some COVID-19 patients’ lymphocytes, which together with the observed *in vitro* IgM-mediated complement activity, suggests that activation of the classical complement pathway, presumably by IgM ALAb, might also play a role *in vivo*. While we did not find IgM ALAb or complement deposition in influenza-challenged patients, it is possible that we looked too early after the infection. It would be interesting to investigate larger cohorts of patients to test whether this phenomenon of IgM ALAb and *in vivo* complement activity is observed in other viral infections besides SARS-CoV-2.

Although we did not find a direct correlation between IgM ALAb and lymphopenia, we did find a correlation between *in vivo* C3b deposition on T lymphocytes and CD4 lymphopenia, suggesting a detrimental effect, specifically on CD4 T cells, of complement deposition, independent of its origin. These data suggest that, similar to what we have previously described in patients with idiopathic CD4 lymphopenia (23), the lymphopenia observed in COVID-19 patients might be potentially caused or exacerbated by complement-activating IgM ALAb. We also found a strong correlation between C3b deposition and C5a plasma levels, which have been associated with disease severity (28). Interestingly, we found that C5a plasma levels correlated stronger with C3b on lymphocytes than with disease severity.

As our study did not include moderately/severely vaccinated patients, we were only able to compare the role of vaccination in mild cases of COVID-19. Within this group, both convalescent and vaccinated patients displayed a similar prevalence of IgM ALAb to acute unvaccinated patients but lower levels of complement deposition. These data suggest that, although infection is sufficient for IgM ALAb generation, avoiding extra inflammation by vaccination might diminish complement deposition on lymphocytes. Although we observed a correlation between IgM ALAb and *in vitro* CDC activity, there was a disconnect between the remaining levels of IgM ALAb and lower levels of *in vivo* complement deposition during convalescence. It is possible that convalescent lymphocytes are more resistant to complement deposition than acute disease lymphocytes because of higher expression levels of complement inhibitory molecules. This might be a consequence of either the modulation of expression or preferential survival after acute disease. In addition, waning inflammation during convalescence might explain the lower complement deposition on convalescent T cells despite the persistent presence of IgM ALAb.

Our current study shows that infection-induced IgM ALAb, together with complement activation, might contribute to lymphopenia and increased disease severity, mostly in unvaccinated COVID-19 patients.

## Data availability statement

The raw data supporting the conclusions of this article will be made available by the authors, without undue reservation.

## Ethics statement

Studies involving humans were approved by the Institutional Review Board of the National Institutes of Health. The studies were conducted in accordance with the local legislation and institutional requirements. The participants provided their written informed consent to participate in this study.

## Author contributions

AP-D: Conceptualization, Methodology, Formal analysis, Project administration, Visualization, Writing –original draft. XL: Investigation, Writing – review & editing. SC: Investigation, Writing – review & editing. AB: Investigation, Writing – review & editing. AL: Resources, Writing – review & editing. AK: Resources. FG: Resources. MJM: Resources, Writing – review & editing. JR: Resources, Writing – review & editing. BE: Resources, Writing – review & editing. EL: Resources, Writing – review & editing. MS: Resources. MM: Resources. GW: Resources, Writing – review & editing. RP: Resources, Writing – review & editing. PK: Resources. IS: Funding acquisition, Conceptualization, Supervision, Writing – review & editing.
