# Supplementary material for: Prevalence of anti-lymphocyte IgM autoantibodies driving complement activation in COVID-19 patients
Source: Front Immunol. 2024 Apr 17;15:1352330. doi: 10.3389/fimmu.2024.1352330 (PMC11061367; doi:10.3389/fimmu.2024.1352330)
Supplement: Supplementary file 1 [file Table_1.docx]

**Supplemental Table S1. Antibodies used for flow cytometry analysis.**

| **Antigen** | **Clone** | **Fluorochrome** | **Supplier** |
| --- | --- | --- | --- |
| C1q | polyclonal | FITC | Dako |
| C3b | 1H8 | PE | Cedarlane |
| CD19 | SJ25C1 | BV510 | BD Biosciences |
| CD19 | HIB19 | APC | BD Biosciences |
| CD3 | SK7 | BUV395 | BD Biosciences |
| CD3 | SK7 | PE-cy7 | BD Biosciences |
| CD4 | SK3 | PE | BD Biosciences |
| CD4 | RPA-T4 | BV605 | BD Biosciences |
| CD4 | RPA-T4 | PE-Cy7 | Thermo Fisher Scientific |
| CD56 | HCD56 | BV421 | Biolegend |
| CD56 | B159 | V450 | BD Biosciences |
| CD8 | SK1 | BV711 | Biolegend |
| CD8 | SK1 | APC | BD Biosciences |
| CD8 | SK1 | BV421 | Biolegend |
| IgG | polyclonal | FITC | Jackson ImmunoResearch |
| IgM | polyclonal | APC | Jackson ImmunoResearch |
| CD55 | IA10 | APC | BD Biosciences |
| CD59 | P282(H19) | BV711 | BD Biosciences |
| CD45RO | UCHL1 | PE-Cy7 | BD Biosciences |
| CD27 | O323 | BV711 | Biolegend |
