# Supplementary material for: Prevalence of anti-lymphocyte IgM autoantibodies driving complement activation in COVID-19 patients
Source: Front Immunol. 2024 Apr 17;15:1352330. doi: 10.3389/fimmu.2024.1352330 (PMC11061367; doi:10.3389/fimmu.2024.1352330)
Supplement: Supplementary file 2 [file Presentation_1.pptx]

## Slide 1
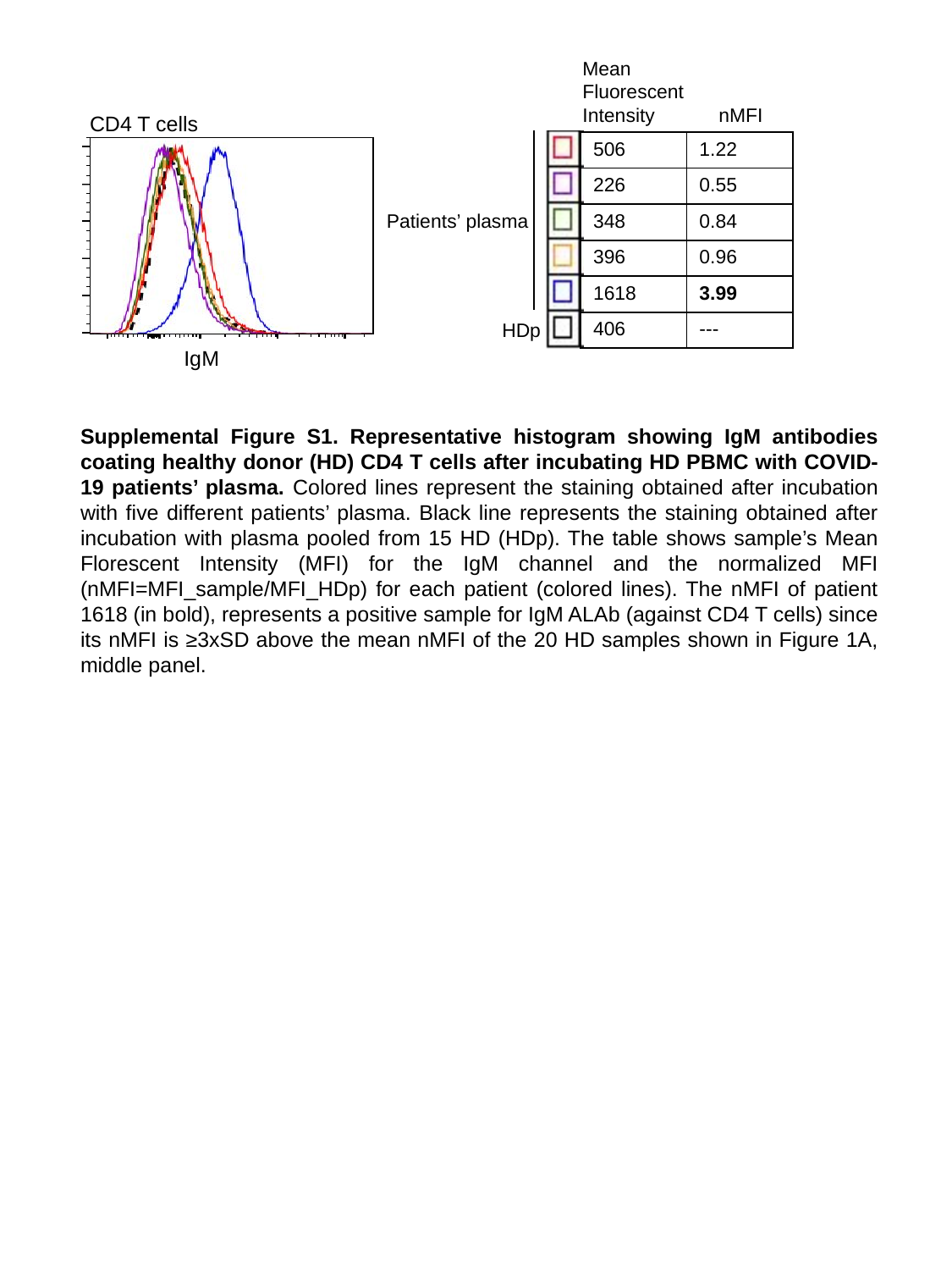

Mean Fluorescent Intensity
nMFI
CD4 T cells
| 506 | 1.22 |
| --- | --- |
| 226 | 0.55 |
| 348 | 0.84 |
| 396 | 0.96 |
| 1618 | 3.99 |
| 406 | --- |
Patients’ plasma
HDp
IgM
Supplemental Figure S1. Representative histogram showing IgM antibodies coating healthy donor (HD) CD4 T cells after incubating HD PBMC with COVID-19 patients’ plasma. Colored lines represent the staining obtained after incubation with five different patients’ plasma. Black line represents the staining obtained after incubation with plasma pooled from 15 HD (HDp). The table shows sample’s Mean Florescent Intensity (MFI) for the IgM channel and the normalized MFI (nMFI=MFI_sample/MFI_HDp) for each patient (colored lines). The nMFI of patient 1618 (in bold), represents a positive sample for IgM ALAb (against CD4 T cells) since its nMFI is ≥3xSD above the mean nMFI of the 20 HD samples shown in Figure 1A, middle panel.

## Slide 2
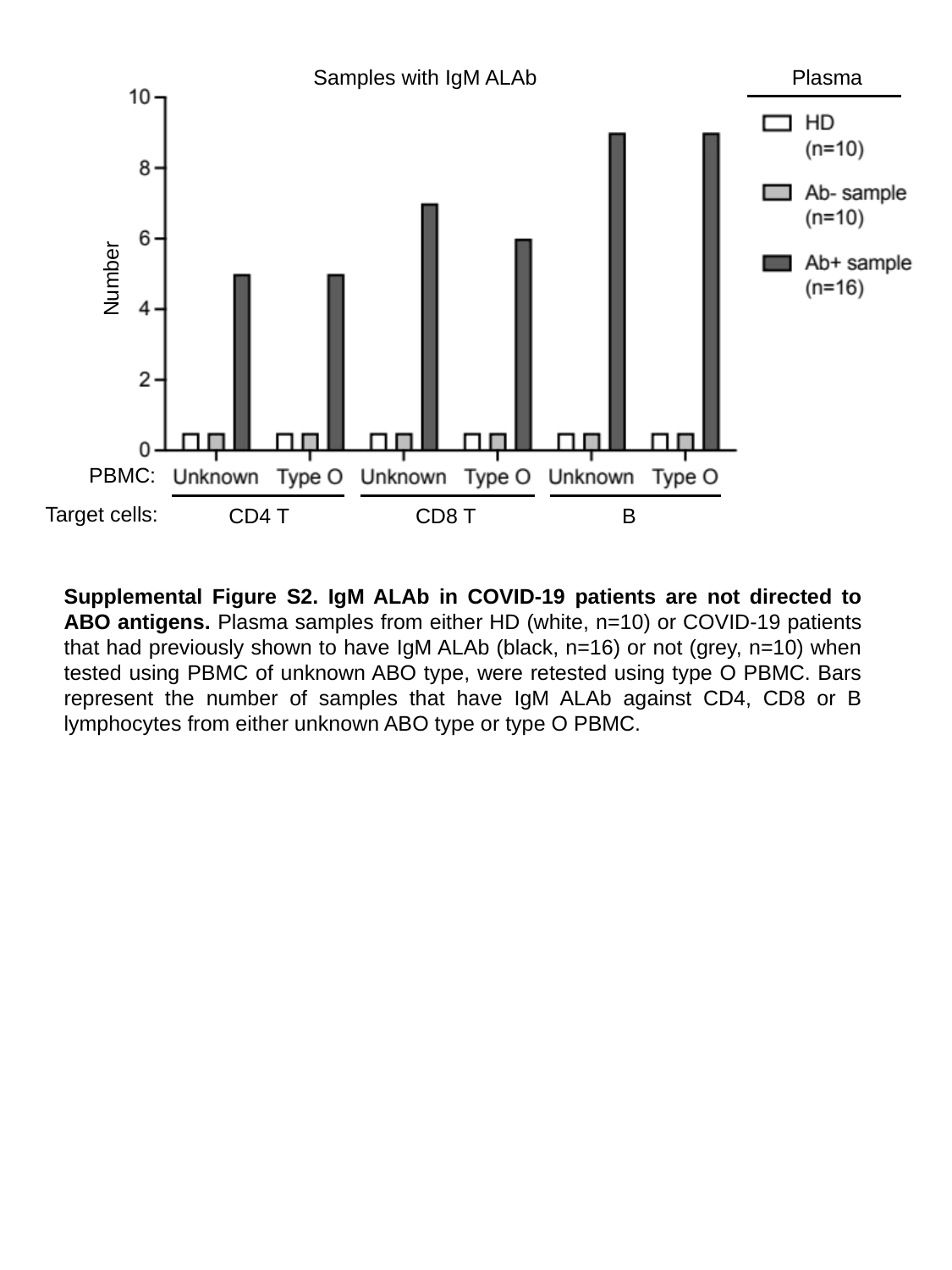

Samples with IgM ALAb
Plasma
Number
PBMC:
Target cells:
CD4 T
CD8 T
B
Supplemental Figure S2. IgM ALAb in COVID-19 patients are not directed to ABO antigens. Plasma samples from either HD (white, n=10) or COVID-19 patients that had previously shown to have IgM ALAb (black, n=16) or not (grey, n=10) when tested using PBMC of unknown ABO type, were retested using type O PBMC. Bars represent the number of samples that have IgM ALAb against CD4, CD8 or B lymphocytes from either unknown ABO type or type O PBMC.

## Slide 3
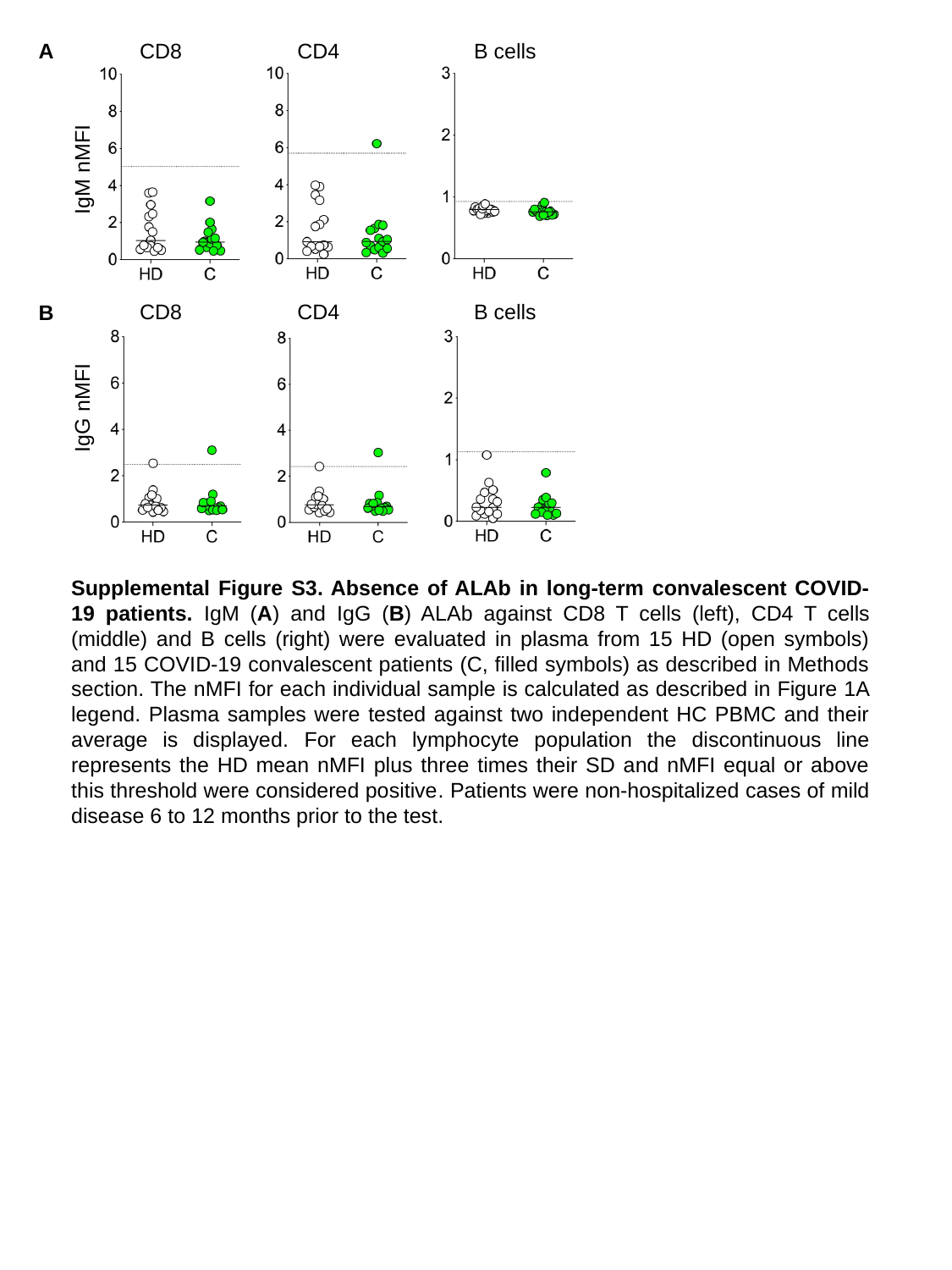

A
CD8
CD4
B cells
IgM nMFI
CD8
CD4
B cells
B
IgG nMFI
Supplemental Figure S3. Absence of ALAb in long-term convalescent COVID-19 patients. IgM (A) and IgG (B) ALAb against CD8 T cells (left), CD4 T cells (middle) and B cells (right) were evaluated in plasma from 15 HD (open symbols) and 15 COVID-19 convalescent patients (C, filled symbols) as described in Methods section. The nMFI for each individual sample is calculated as described in Figure 1A legend. Plasma samples were tested against two independent HC PBMC and their average is displayed. For each lymphocyte population the discontinuous line represents the HD mean nMFI plus three times their SD and nMFI equal or above this threshold were considered positive. Patients were non-hospitalized cases of mild disease 6 to 12 months prior to the test.

## Slide 4
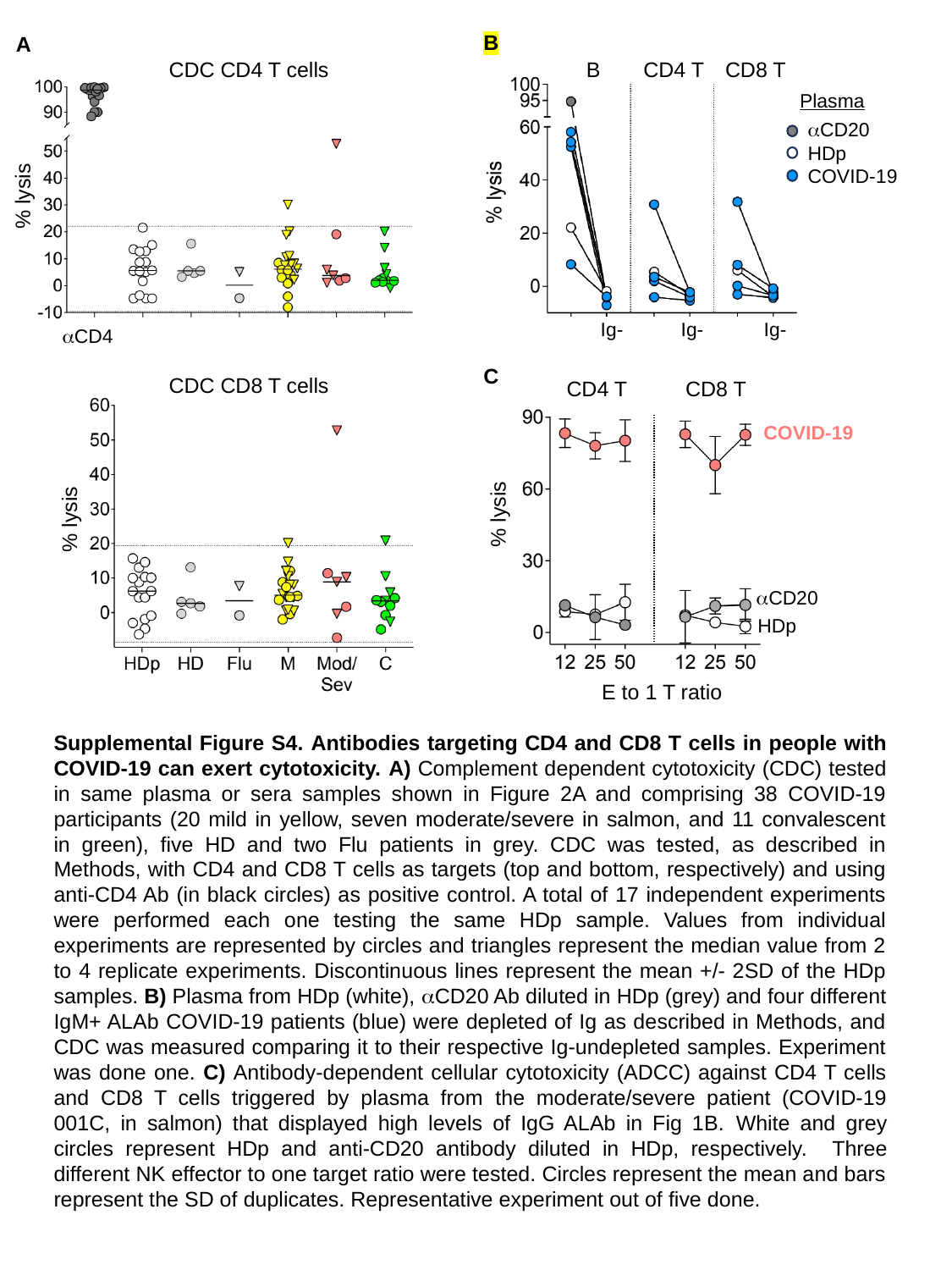

B
A
CDC CD4 T cells
B
CD4 T
CD8 T
Plasma
aCD20
HDp
COVID-19
Ig-
Ig-
Ig-
% lysis
aCD4
C
CDC CD8 T cells
CD4 T
CD8 T
COVID-19
% lysis
% lysis
aCD20
HDp
E to 1 T ratio
Supplemental Figure S4. Antibodies targeting CD4 and CD8 T cells in people with COVID-19 can exert cytotoxicity. A) Complement dependent cytotoxicity (CDC) tested in same plasma or sera samples shown in Figure 2A and comprising 38 COVID-19 participants (20 mild in yellow, seven moderate/severe in salmon, and 11 convalescent in green), five HD and two Flu patients in grey. CDC was tested, as described in Methods, with CD4 and CD8 T cells as targets (top and bottom, respectively) and using anti-CD4 Ab (in black circles) as positive control. A total of 17 independent experiments were performed each one testing the same HDp sample. Values from individual experiments are represented by circles and triangles represent the median value from 2 to 4 replicate experiments. Discontinuous lines represent the mean +/- 2SD of the HDp samples. B) Plasma from HDp (white), aCD20 Ab diluted in HDp (grey) and four different IgM+ ALAb COVID-19 patients (blue) were depleted of Ig as described in Methods, and CDC was measured comparing it to their respective Ig-undepleted samples. Experiment was done one. C) Antibody-dependent cellular cytotoxicity (ADCC) against CD4 T cells and CD8 T cells triggered by plasma from the moderate/severe patient (COVID-19 001C, in salmon) that displayed high levels of IgG ALAb in Fig 1B. White and grey circles represent HDp and anti-CD20 antibody diluted in HDp, respectively. Three different NK effector to one target ratio were tested. Circles represent the mean and bars represent the SD of duplicates. Representative experiment out of five done.

## Slide 5
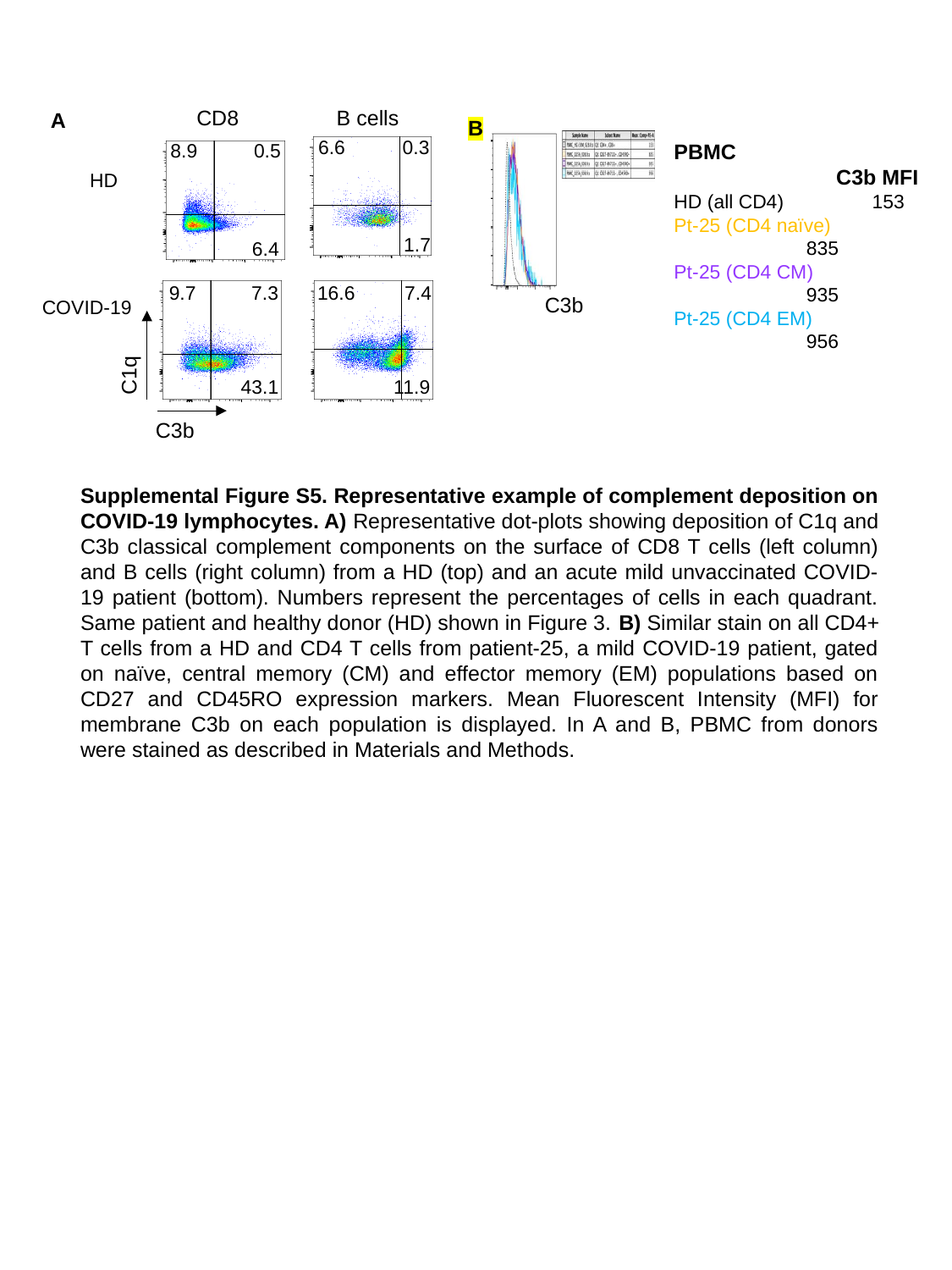

CD8
B cells
A
B
6.6
0.3
1.7
8.9
0.5
6.4
PBMC		 C3b MFI
HD (all CD4)	 153
Pt-25 (CD4 naïve)	 835
Pt-25 (CD4 CM)	 935
Pt-25 (CD4 EM)	 956
HD
9.7
7.3
43.1
16.6
7.4
11.9
C3b
COVID-19
C1q
C3b
Supplemental Figure S5. Representative example of complement deposition on COVID-19 lymphocytes. A) Representative dot-plots showing deposition of C1q and C3b classical complement components on the surface of CD8 T cells (left column) and B cells (right column) from a HD (top) and an acute mild unvaccinated COVID-19 patient (bottom). Numbers represent the percentages of cells in each quadrant. Same patient and healthy donor (HD) shown in Figure 3. B) Similar stain on all CD4+ T cells from a HD and CD4 T cells from patient-25, a mild COVID-19 patient, gated on naïve, central memory (CM) and effector memory (EM) populations based on CD27 and CD45RO expression markers. Mean Fluorescent Intensity (MFI) for membrane C3b on each population is displayed. In A and B, PBMC from donors were stained as described in Materials and Methods.
